# Public support for government regulatory interventions for overweight and obesity in Australia

**DOI:** 10.1186/s12889-018-5455-0

**Published:** 2018-04-18

**Authors:** Emma Sainsbury, Chelsea Hendy, Roger Magnusson, Stephen Colagiuri

**Affiliations:** 1 The Boden Institute of Obesity, Nutrition, Exercise & Eating Disorders, Charles Perkins Centre, The University of Sydney, Camperdown, 2006 Australia; 2 Sydney Law School, The University of Sydney, Camperdown, 2006 Australia

**Keywords:** Overweight, Obesity, Australia, Regulation, Public opinion, Advertising, Sport sponsorship, Taxation

## Abstract

**Background:**

There is growing recognition among public health circles of the need for regulatory action for overweight and obesity, but there has been limited research into whether the Australian public supports government intervention. This study aimed to determine the level of public support for food-related regulations for obesity, and to assess the determinants of support.

**Methods:**

A nationally representative sample of Australian adults (*n* = 2011) was recruited by market research company Online Research Unit to complete an online survey. The survey measured respondents’ perception of the obesity problem in Australia, and level of agreement on a 5-point Likert scale (strongly disagree to strongly agree) with proposed regulations in three domains; advertising, sponsorship of children’s sport, and taxation. Binary logistic regression models were run to examine the association between demographic variables and support for regulation.

**Results:**

The majority of respondents (92.5%) considered overweight and obesity to be a somewhat or very serious problem in Australia, and almost 90% felt there should be at least some government regulation to protect the public. Respondents agreed that the government should regulate food and beverage advertising (69.5%), with strongest support for restricting unhealthy food advertising to children (78.9%). There was lower support for prohibiting unhealthy food and beverage company sponsorship of children’s sport (63.4% agreement), and for taxing sugar-sweetened beverages (54.5%), although the majority were still in favour. Support for fiscal policies slightly increased if revenue was to be used for health purposes. Females and tertiary educated respondents showed stronger agreement with proposed regulations (*p* < 0.05).

**Conclusions:**

The survey findings suggest the majority of the Australian population recognises obesity to be a serious health problem, and support government regulation of the food environment as a population-level preventative strategy.

**Electronic supplementary material:**

The online version of this article (10.1186/s12889-018-5455-0) contains supplementary material, which is available to authorized users.

## Background

Rates of overweight and obesity in Australia have been steadily climbing. In 2014–15, 63.4% of adults and 27.4% of children were classified as overweight or obese [1], with models predicting significant increases in the prevalence of severe obesity (BMI > 35) by 2025 [2]. The social, health and economic impacts of overweight and obesity are significant, including increased risk of non-communicable diseases such as cancer, cardiovascular disease and type 2 diabetes, and reduced quality of life [3]. The financial cost of overweight and obesity was estimated at $58.2 billion in 2008, including productivity costs, government subsidies, and loss of wellbeing [4]. A growing literature recognizes the impact that changes in the food environment, including the increased availability, affordability and marketing of energy-dense, nutrient-poor foods and beverages are having on dietary behaviours and rates of overweight and obesity [5–7]. Poor diet is now identified as the leading preventable behavioural risk factor for global burden of disease, overtaking tobacco use [8].

From both a health and economic standpoint, there is an urgent need to improve the healthiness of diets and prevent overweight and obesity. The World Cancer Research Fund International developed the NOURISHING framework to highlight the various policy actions governments should take under three domains; the food environment, food system and behaviour change [9]. To date, Australian governments at the state and federal level have prioritised behaviour change strategies, investing public health dollars into education campaigns and settings-based programs for obesity prevention at the level of the individual. What regulatory action the government has taken to promote a healthier food environment and improve population-level dietary behaviours has been uncoordinated between the states and territories, and has had limited success in preventing the continued rise in rates of overweight and obesity [10–12]. New South Wales (NSW), Australian Capital Territory (ACT), Queensland and South Australia have introduced mandatory kilojoule labelling in fast food restaurants, although other states are yet to take action [9]. The Australian Government also introduced the health star rating scheme in 2014, a front-of-pack labelling system that provides a quick and easy way for consumers to select healthier packaged foods [13]. This scheme is still voluntary for the food industry, and it is currently estimated to appear on only 60% of packaged foods [14]. At the Commonwealth level, Baker and colleagues point to a range of factors which jointly contribute to the lack of priority given to obesity prevention [15]. These factors include the substantial political influence wielded by food companies and industry associations, assisted by their ease of access to policy elites, their importance to Australia’s economy, and their adoption of voluntary and self-regulatory instruments [15, 16]. Broader contextual factors include the dominance of a political culture in which regulatory actions are framed as “nanny state” interventions that inappropriately seek to displace parental and personal responsibility. Crammond and colleagues also point to the need to subject new policy proposals to regulatory impact analysis and to show that the benefit of each individual proposal outweighs the cost to business [17].

Policy and regulatory controls, including taxation of unhealthy foods and beverages and restrictions on unhealthy food advertising to children have been recognised by the World Health Organization as priority areas for action [18, 19], and recommendations for government intervention in Australia are building [10, 20, 21]. There is evidence to support the effectiveness of taxes and advertising restrictions for improving dietary behaviours within countries where these policies have been adopted. Mexico’s 1 peso per litre tax on sugar-sweetened beverages (SSBs) resulted in a 5.5% decline in purchases of taxed beverages in 2014, and a 9.7% decline the following year, with an average 7.6% decline over two years [22]. In Hungary, the introduction of a tax on junk food in 2011 resulted in an estimated 3.4% reduction in consumption of processed foods, and a 1.1% increase in consumption of unprocessed foods [23]. Further, bans on commercial food advertising to children in Quebec resulted in a reduction of US$88 million spent on fast food during 2010, and a reduction of 13.4–18.4 billion fast food calories consumed by French-speaking households [24]. Although taxation and advertising restrictions have not yet been implemented in Australia for obesity prevention, they have been successfully implemented in the field of tobacco control. Between 1991 and 2016, daily smoking in people aged 14 years and older fell from 24.3% to 12.2% through the combined impact of increases in the tobacco excise, the introduction of graphic health warnings on tobacco packages, comprehensive bans on tobacco advertising, including tobacco plain packaging legislation, and bans on smoking in enclosed public places and outdoor public areas [25]. Tobacco taxation has been consistently recognised as the most effective public health policy for reducing tobacco consumption in Australia [26].

Although evidence of regulatory effectiveness is important for informing obesity prevention efforts, the acceptability of these measures to the food industry and to the wider public are significant variables affecting their likely implementation. Governments are sensitive to public opinion and unlikely to take action in the absence of significant levels of public support [27]. Studies in the United States (US) [28], United Kingdom [29] and across the European Union [30, 31] have reported strong public support for food labelling policies and restrictions on unhealthy food advertising to children, with the least support recorded for price-raising policies such as junk food and SSB taxes. Within Australia, there have been few large-scale surveys comparing support for different regulatory approaches. Morley et al. interviewed 1511 adults from across Australia who were the main grocery buyer in their household, revealing up to 90% support for restricting unhealthy food advertising to children, and 71% support for a tax on unhealthy foods and controls on sponsorship of sport by food companies [32]. Similarly, a survey of 2147 Western Australian adults reported 84% of respondents to be in favour of advertising restrictions [33]. These studies were not conducted on a nationally representative sample, and therefore may not accurately reflect Australia’s attitudes towards regulatory approaches to obesity prevention. Other Australian studies have been conducted on small samples [34–38], have targeted a niche group of participants [34, 39, 40], or have measured support for only one form of regulation [41, 42]. In addition, evidence about which demographic characteristics are associated with greatest policy support have been inconclusive and variable between studies and more data are needed if advocacy efforts are to be appropriately targeted. This study aimed to: (1) determine the level of public support in Australia for regulation of the food environment; and (2) assess the demographic characteristics that determine support for regulatory interventions to prevent overweight and obesity. The results of this study will be used to inform future strategies to influence regulatory action by the Australian government and contribute to more strategic and effective advocacy by the public health sector.

## Methods

### Ethics approval

This study was approved by the Human Research Ethics Committee at the University of Sydney [No. 2017/202]. Before commencing the study, participants were asked to read a Participant Information Sheet and an informed consent statement. Participants were required to indicate their consent by clicking ‘Yes’ on an electronic consent form before the survey could begin. Participants were informed about their right to exit the survey at any time, and all participant data were analysed and reported anonymously.

### Participant recruitment

An online market research survey was administered to a cross-sectional sample of Australian residents aged ≥18 years in May 2017. Market research company Online Research Unit (ORU) was contracted to recruit participants and distribute the survey. Participants were recruited using both online (email, internet) and offline (telephone, print and postal) methods to ensure broad demographic representation, and to limit recruitment of professional survey respondents. ORU used stratified sampling to ensure respondents were nationally representative for age, gender and location. The survey was run over 12 days until the minimum 2000 responses were received. Participants were rewarded points worth a nominal value ($2.00) upon completion of the survey.

### Survey design

The survey, hosted through secure web application Redcap, measured respondents’ perception of the obesity problem in Australia, the responsibility of governments, and level of agreement on a 5-point Likert scale (strongly disagree to strongly agree) with proposed government regulations (Additional file 1). Proposed regulatory approaches included restrictions on food and beverage advertising, prohibiting food and beverage sponsorship of children’s sport and food and beverage taxation. Participants were also asked to respond to seven demographic questions including gender, age, education level, postcode, weight, height and parental status. The survey questions were developed in consultation with an advisory group of public health experts experienced in market research surveys.

A pilot version of the survey was conducted with 166 respondents and the data analysed to measure the scale reliability and validity of the survey tool. Principal components analysis was carried out using IBM Windows SPSS version 22.0 [43] on the questions measuring support for proposed government regulations (question 3–13). Two components recorded initial eigenvalues greater than 1.0, explaining 76.1% of the variance: (1) support for advertising and marketing restrictions; and (2) support for food and beverage taxation. Internal consistency within each component was evaluated using Cronbach’s α. Both components had high internal consistency, with Cronbach’s α of 0.92 and 0.95 for components 1 and 2 respectively. The effect of removing individual questions on the Cronbach’s α was calculated and no redundant questions were identified. Only one error was detected during data cleaning (question 15 was missing an age-range category) and this question was corrected before the official survey launch. Detailed results of the pilot survey are available from the corresponding author on request.

### Data analysis

Survey data were exported from Redcap and analysed using IBM Windows SPSS version 22.0 [43]. Only completed surveys were eligible for analysis; surveys with one or more fields missing were coded as a partial response. The Australian Bureau of Statistics (ABS) 2011 Socio-economic Indexes for Areas (SEIFA) Index of Relative Socioeconomic Advantage and Disadvantage was used to group participants into socioeconomic status (SES) tertiles based on postcode [44]. Weight and height data were used to calculate BMI, and participants were coded as underweight (< 18.5 kg/m^2^), healthy weight (18.5–24.9 kg/m^2^), overweight (25.0–29.9 kg/m^2^), or obese (≥ 30 kg/m^2^). BMIs outside the range 14–60 kg/m^2^ were coded as invalid responses. Post-stratification weights were applied to ensure sample characteristics matched the general population for all demographic variables, based on ABS 2011 Census data. The results reported in this paper are based on weighted data; the unweighted results are available on request from the authors.

**Table 1:** Demographic characteristics of survey participants (*n* = 2011)

Characteristic	Number	Unweighted %	Weighted *%*
Gender			
Female	1000	49.7	49.9
Male	1010	50.2	50.1
Other	1	0.0	0.0
Age (years)			
18–24	127	6.3	5.2
25–34	292	14.5	11.3
35–44	370	18.4	16.1
45–54	312	15.5	16.5
55+	910	45.3	50.7
Education level			
Did not complete school	183	9.1	25.0
Year 12 high school	376	18.7	18.0
TAFE/trade/diploma	642	31.9	32.0
University degree or above	810	40.3	25.0
Location			
Victoria	586	29.1	28.9
NSW	584	29.0	28.3
Queensland	405	20.1	20.6
South Australia	182	9.1	9.6
Western Australia	171	8.5	8.5
Tasmania	54	2.7	2.9
ACT	24	1.2	0.9
Northern Territory	5	0.2	0.3
Parental status			
Children	1302	64.7	66.5
No children	679	33.8	32.2
Prefer not to disclose	30	1.5	1.3
Socioeconomic status			
Mean SEIFA score (range)	1005.4 (627–1164)		
Tertile 1	672	33.4	36.5
Tertile 2	674	33.5	34.4
Tertile 3	665	33.1	29.1
BMI			
Mean BMI (kg/m^2^) (range)	27.0 (14.7–59.5)		
Underweight	56	2.8	2.5
Healthy weight	731	36.4	33.2
Overweight	649	32.3	31.5
Obese	506	25.2	29.1
Invalid response	69	3.4	3.7

Tertile 1 indicates respondents from areas of lowest socioeconomic advantage and Tertile 3 indicates respondents from areas of highest socioeconomic advantage

Survey questions were entered into SPSS as separate variables and participant responses scored on an ordinal scale, with a score of 1 allocated for ‘strongly disagree’, through to 5 for ‘strongly agree’. Descriptive and cross-tabulation statistics were run to describe the demographic characteristics of participants and measure overall agreement (strongly or somewhat) with each proposed regulation. Outcomes were then dichotomized as “agree” (combining “strongly agree” and “somewhat agree” responses) or “disagree” (combining “strongly disagree” and “somewhat disagree” responses) for questions 1–13, and binomial logistic regression models were run to examine the association between demographic variables (gender, age, location, education level, SES, BMI and parental status) and support for regulation (Additional file 2). A significance level of *p* < 0.05 was accepted throughout.

**Table 2:** Respondent agreement with proposed regulatory approaches for overweight and obesity prevention (n = 2011)

Question	Very/somewhat serious (%)	Not too/not at all serious (%)	Don’t know (%)
How serious do you consider the problem of overweight and obesity in Australia?	92.5	6.1	1.4
	A great deal/some/a little (%)	None at all (%)	Don’t know (%)
How much government regulations do you think there should be around protecting people from overweight and obesity in Australia?	86.1	9.4	4.4
	Agree (%)	Neither agree nor disagree (%)	Disagree (%)
Government regulations should restrict advertising of unhealthy foods and beverages on television during times when children are watching	78.9	12.2	8.9
Government regulations should restrict advertising of unhealthy foods and beverages to children on the internet	75.8	13.9	10.2
Government regulations should restrict advertising of unhealthy foods and beverages at sporting events	71.4	17.0	11.6
Government regulations should restrict advertising of unhealthy foods and beverages in public spaces (e.g. bus stops, train stations, roadside)	70.3	18.3	11.4
The government should regulate food and beverage advertising	69.5	18.0	12.6
Government regulations should prohibit sugar-sweetened beverage companies from sponsoring children’s sporting organisations, teams and events	63.4	18.6	18.0
Government regulations should prohibit fast food companies from sponsoring children’s sporting organisations, teams and events	58.7	21.6	19.7
The government should introduce a tax on sugar-sweetened beverages and use the money raised to fund health services and programs to reduce overweight and obesity	57.2	17.4	25.5
The government should introduce a tax on unhealthy foods, and use the money raised to fund health services and programs to reduce overweight and obesity	54.6	18.6	26.9
The government should introduce a tax on sugar-sweetened beverages	54.5	16.9	28.5
The government should introduce a tax on unhealthy foods	48.9	19.7	31.3

Descriptive results are based on data weighted for education level. The “% agree” category represents respondents who “somewhat agree” or “strongly agree” with the proposed regulatory measures. The “% disagree” category represents respondents who “somewhat disagree” or “strongly disagree” with the proposed regulatory measures

## Results

### Participant characteristics

A total of 2135 participants accessed the survey, with 2011 complete and 124 partial responses. Table 1 shows the demographic characteristics of participants. The gender, age, location, BMI and parental status of participants were reflective of the Australian population, based on ABS 2011 Census Data. Survey participants were more highly educated than the general population, with 72.2% completing a tertiary education qualification (University degree, Technical and Further Education [TAFE], trade or diploma) compared to only 57.0% of the Australian population. Data were weighted for education level in analysis.

### Perception of the overweight and obesity problem in Australia

The majority of participants (92.5%) considered overweight and obesity to be a somewhat or very serious problem in Australia, with 5.1% perceiving it as not too serious and 1.0% perceiving it as not at all serious (Table 2). Males were significantly less likely than females to consider overweight and obesity to be a serious issue [Odds ratio (OR) 0.46, 95% 0.24–0.85 *p* = 0.01] (Additional file 2). Parental status also influenced opinions, as participants without children were significantly less likely than participants with children to agree that overweight and obesity is a serious problem in Australia [OR 0.43, 95% CI 0.23–0.82, *p* = 0.01]. Very few participants felt there was no need for government regulation to address overweight and obesity (9.4%), with the remaining participants believing there should be a little (20.5%), some (43.5%) or a great deal (22.1%) of regulation. University educated participants were significantly more likely than those who did not complete school to feel more government regulation was needed [OR 1.68, 95% CI 1.24–2.28, *p =* 0.001].

### Public support for regulation of food and beverage advertising

Food and beverage advertising regulations were the best supported of the policy options proposed in the questionnaire (Table 2). Support was highest for restrictions on unhealthy food advertising directed at children on television (78.9%) and the internet (75.8%). Females were consistently more likely than males to agree that the government should regulate unhealthy food and beverage advertising, irrespective of the medium (*p* < 0.05) (Additional file 2). Support for advertising restrictions decreased as SEIFA score increased, with those in the highest SES tertile (i.e. from areas of highest socioeconomic advantage) significantly less likely than those in the lowest SES tertile to support government regulation of unhealthy food and beverage advertising on television (OR 0.50, 95% CI 0.33–0.77, *p* = 0.001], the internet [OR 0.53, 95% CI 0.36–0.79, *p* = 0.002], in public spaces [OR 0.52, 95% CI 0.36–0.76, *p =* 0.001] and at sporting events [OR 0.50, 95% CI 0.34–0.71, *p* < 0.001]. Respondents with a tertiary education qualification were also more likely to support proposed advertising restrictions (*p* < 0.05).

### Public support for regulation of children’s sport sponsorships

Fifty-nine percent of participants agreed with prohibiting fast food company sponsorship of children’s sport, and 63.4% supported prohibitions on SSB company sponsorship (Table 2). As seen in Additional file 2, education level was a consistent predictor of support, with tertiary educated respondents significantly more likely than those who did not complete high school to agree with government regulation of children’s sport sponsorships (*p* < 0.001). Males were also significantly less likely than females to support prohibitions on fast food company sponsorship [OR 0.65, 95% CI 0.51–0.83, *p* < 0.001] and SSB company sponsorship of children’s sport [OR 0.68, 95% CI 0.53–0.87, *p =* 0.002].

### Public support for food and beverage taxation

There was least support among survey respondents for a tax on unhealthy foods (48.9%) and SSBs (54.5%); however, there was a slight increase in regulatory support if the revenue generated from the tax is used for health purposes (Table 2). Age was a predictor of attitudes towards regulation, with younger respondents (18–24 years) more supportive of a hypothecated tax (*p* < 0.05) (Additional file 2). Respondents who completed a university degree were significantly more likely than those who did not complete high school to support taxation of unhealthy foods [OR 2.27, 95% CI 1.63–3.15, *p* < 0.001] and SSBs [OR 2.74, 95% CI 1.95–3.84, *p* < 0.001]. Respondents without children were significantly less likely to support government taxation of unhealthy foods [OR 0.78, 95% CI 0.62–0.99, *p =* 0.04], although this relationship was not observed for SSB taxation.

## Discussion

Given rising rates of overweight and obesity in Australia, public health experts have called for greater government accountability and regulation of the food environment as a population-level preventative strategy [10, 20]. The results of this survey indicate that Australians overwhelmingly recognise overweight and obesity as a serious health problem, with close to 90% agreeing that at least some government regulation is required. When considering what kinds of regulatory actions should be taken, there was strongest support for restrictions on the advertising of unhealthy foods and beverages, with less support for taxation of unhealthy foods and SSBs. Demographic characteristics of participants were a determinant of support, with females and tertiary educated individuals more supportive of government regulation.

### Regulation of food and beverage advertising

The results of this survey demonstrate that there is substantial public support for regulations to reduce exposure to unhealthy food and beverage advertising in Australia, in particular broadcast advertising. Nearly four in five respondents believed that the government should restrict unhealthy food and beverage advertising during times when children are watching television, and three in four supported restrictions on unhealthy food and beverage advertising to children on the internet. Earlier Australian studies reported slightly higher levels of support for restrictions on unhealthy food advertising directed at children (83%), although this was likely due to sampling differences as the studies predominantly surveyed females [32, 33]. There is a growing body of literature highlighting the direct causal link between advertising exposure and the taste preferences, purchasing preferences, and consumption of unhealthy food by children [6, 45–48], and parental concerns are rising about the high volume of unhealthy food advertising directed to children on television [49, 50]. As community awareness about the negative influence of food advertising builds, it may be expected that support for regulation will continue to rise.

The results of this survey also indicate strong support (> 70% agreement) for restrictions on unhealthy food advertising at sporting events and in public areas such as transit stops. Advertisements in public settings have the potential to significantly influence the dietary behaviours of both children and adults, given that these areas are highly frequented and the nature of the viewing is non-discretionary. Recent Australian research demonstrates that over 80% of food and beverage advertising at train stations and in the areas surrounding schools are for unhealthy products [51–53], suggesting that government regulation of these settings is also warranted.

### Regulation of children’s sport sponsorship

Nearly two out of three respondents supported regulations banning fast food and SSB companies from sponsoring children’s sport (63.4% support overall); a similar result to previous Australian surveys [39, 42]. The community appreciates that sporting clubs require financial assistance, and that sponsorship can assist in subsidising cost of participation and reducing equipment and resource costs [39]. A public survey conducted by the Victorian Health Promotion Foundation in 2009 (*n* = 1500) found that 53% of respondents opposed junk food sponsorship of community sports clubs, but support for removing sponsorship increased to 81% if the government were to replace lost revenue [54]. The results may also reflect the parental perception that children are less influenced by sponsorship of amateur sport compared with elite sport sponsorship [39], even though studies have shown that children have high brand recall and positive attitudes towards food and beverage sponsorship companies [55]. Parents were as likely as non-parents to agree with the proposed regulations, an unexpected but promising finding suggesting broad community concern for the health of children.

### Taxation of unhealthy foods and beverages

In line with previous local and international research, taxes on unhealthy food and SSBs were less well-supported than advertising restrictions [56–58], although support was slightly higher if the revenue was used for public health purposes. There is continuing public debate between health experts, policymakers, and the food and beverage industry about the merits of a tax on unhealthy foods and beverages. Key anti-tax messages that have been circulated by the food and beverage industry include the potential loss of revenue and employment, the regressive nature of the tax for lower SES groups, and the ineffectiveness of taxes in improving unhealthy dietary behaviours [59]. In addition, taxes are commonly framed as “nanny-state” interventions that restrict individual freedom of choice [60]. A recent study which assessed public opinion in the US towards arguments used in tax debates showed that the public more strongly agreed with anti-tax messages compared with pro-tax messages [61], indicating a need to re-frame and strengthen the pro-tax messages that are disseminated.

Despite taxation being the least favoured policy option, there was still considerable public support for a tax on SSBs, particularly if the money raised is used for health-related purposes (57.2%). Consumption of SSBs in Australia is high [62], and there is an established association between rising consumption of SSBs and rising rates of obesity, type 2 diabetes and dental caries [63, 64]. What constitutes a sugar-sweetened beverage is easy to define, whereas a tax on unhealthy foods is more ambiguous, and could potentially have unintended health effects due to patterns of food substitution in response to the tax [65].

### Determinants of public support for regulation

The results of this survey highlight the influence of demographic factors, in particular gender and education level, on support for government regulation. Despite no significant between-group difference in the perceived severity of the obesity problem, respondents with a tertiary education were significantly more supportive of all proposed regulations, in line with existing literature [49, 57, 66, 67]. It is hypothesised that this is due to educated respondents having a greater awareness and understanding of the environmental influences on health. While it was expected that higher SES groups would also be more supportive of government regulation due to the known correlations between education level and SES, this was not observed, with lower SES respondents more supportive of advertising restrictions.

Females had a tendency to be more supportive of regulation of the food environment, whether because they are more health conscious or because they are more likely to be the household’s primary grocery buyer, with greater appreciation of the challenges of purchasing healthy food. Younger respondents aged 18–24 years were also significantly more likely to support taxation of SSBs and unhealthy foods compared with older adults, particularly if the money was to be used for health-related purposes. Our results are consistent with those of Rivard et al., who observed greater support for an SSB tax among younger respondents [66]. This age-group is the largest consumer of SSBs in Australia [62], and would be expected to benefit most from a tax.

### Limitations

Unlike other studies which examined determinants of support for obesity prevention policies, this survey did not explore the public’s beliefs about the causes of overweight and obesity. Previous literature has suggested that those who perceive environmental factors to be the biggest driver of overweight and obesity are significantly more supportive of government regulation compared to those who attribute it to individual choice [28]. Further, this survey did not explore why certain regulations were not supported (e.g. whether viewed as intrusive, or ineffective in changing behaviours). The grouping of participants into BMI categories was based on self-reported height and weight. Given the tendency for people to underreport these values, we are unable to accurately assess whether BMI is associated with support for regulation.

### Implications for future action

In Australia, the federal government’s constitutional powers enable it to regulate broadcast (including television and radio) and digital advertising, although state governments retain regulatory power over outdoor advertising – a prime setting for unhealthy food and beverage advertising [51]. Under the co-regulatory framework governing broadcasting regulation, the Australian Communication and Media Authority (ACMA) is legally authorised to register and monitor codes of practice developed in conjunction with industry groups, a number of which contain controls on advertising [68]. One model for regulating broadcast advertising would therefore be to introduce a code imposing appropriate constraints on unhealthy food and beverage advertising that is registered with the ACMA, becoming part of the licence conditions for broadcasting licensees. However, due to the variety of advertising formats and platforms available, regulation of advertising on the internet and on mobile communications is likely to require controls to be extended beyond broadcast licensees. For example, federal legislation already prohibits the publication of tobacco advertisements in Australia, including advertisements that are accessed or disseminated through the internet, computers, mobile devices and other electronic devices [69].

Currently in Australia there is no policy or regulation that limits or restricts the types of companies allowed to sponsor children’s sport. Before prohibitions on food company sponsorship can be acceptably introduced by the government, there needs to be consideration of how children’s sports clubs will continue to be funded. Pettigrew et al. measured what sponsors the public would consider acceptable at children’s sport, identifying health agencies, sporting goods companies, banks/finance companies and bottled water companies as suitable options [42]. Alternatively, Kelly et al. proposed the establishment of an intermediary Sport Sponsorship Fund that could manage the collection and distribution of corporate funding for sports clubs; a strategy that was well supported by parents [70]. State government health agencies could also work as funding bodies for sporting clubs: the ACT, South Australia, Victoria and Western Australia adopted this approach following the introduction of the Federal Tobacco Advertising Prohibition Act in 1992 [71].

More than half of respondents in our survey supported a tax on sugar-sweetened beverages, and nearly half supported a tax on unhealthy foods. There are a number of tax models for consideration by federal government; specific excise taxes being the most widely accepted and used [19]. An excise tax on SSBs, if implemented at a tiered rate as in England, has the potential to reduce sugar intake, generate government revenue, educate consumers and drive the reformulation of beverages [72]. Although one in four survey respondents disagreed with introducing a tax on unhealthy foods and beverages, literature has shown that support for public health policies such as smoking bans [73], tobacco taxation [26] and mandatory seat-belt legislation [74] can increase post-policy implementation. A recent study measuring public acceptance of the SSB tax in France (*n* = 1996) also reported an increase in support post-policy implementation [57]. Public acceptance of taxes was shown to increase if the money raised is used for health purposes, so marketing taxes as a revenue-raising tool rather than a preventative health initiative is likely to build both public and political support. Additionally, more advocacy work is needed to educate the public on the positive health, economic and social impacts of SSB taxes, as a significant number of survey respondents appeared to have no opinion on the issue (17.4% neither agree nor disagree). Advocacy and education needs to be carefully targeted to the right demographic groups, in particular males and those without a tertiary education.

## Conclusion

The findings of this study indicate that there is strong recognition of the obesity problem in Australia, and widespread public support for government regulations to improve the healthiness of the food environment. Of the regulatory options proposed, there was strongest support for restricting unhealthy food and beverage advertising to children on TV and the internet, and at sporting events and in public places. Despite fiscal interventions being less favoured, the majority of survey respondents still supported a tax on SSBs. The results indicate that policymakers are out of step with public opinion on the necessity for government intervention, and that regulatory action should be taken to ensure that public trust and confidence is maintained. Education and advocacy efforts designed to strengthen public support should be targeted towards males and those without a tertiary education.

## Additional files

Additional file 1: Online market research survey. (DOCX 24 kb)

Additional file 2: Binomial logistic regression predicting responses to regulatory approaches based on demographic characteristics. (DOCX 61 kb)

## Abbreviations

ABS: Australian Bureau of Statistics
ACMA: Australian Communication and Media Authority
ACT: Australian Capital Territory
NSW: New South Wales
OR: odds ratio
ORU: Online Research Unit
SEIFA: socio-economic indexes for areas
SES: socioeconomic status
SSB: sugar-sweetened beverage
US: United States
