# Supplementary material for: Public support for government regulatory interventions for overweight and obesity in Australia
Source: BMC Public Health. 2018 Apr 18;18:513. doi: 10.1186/s12889-018-5455-0 (PMC5907362; doi:10.1186/s12889-018-5455-0)
Supplement: Supplementary file 1 — Online market research survey. (DOCX 24 kb) [file 12889_2018_5455_MOESM1_ESM.docx]

**Additional file 1. Online market research survey**

INTRODUCTION

Dear Sir/Madam,

Thank you for agreeing to participate in this survey which aims to:

**Explore the opinions and attitudes of the Australian public towards regulatory approaches for reducing overweight and obesity.**

Please read the Participant Information Sheet before commencing the survey [attach PIS]

**CONSENT**

**Study name: Market survey on regulatory approaches to reduce overweight and obesity in Australia**

In giving my consent, I state that: 

1. I understand the purpose of the study, what I will be asked to do, and any risks/benefits involved

2.  I have read the Participant Information Statement and have been able to discuss my involvement in the study with researchers if I wished to do so

3.  The researchers have answered any questions that I had about the study and I am happy with the answers

4.  I understand that being in the study is completely voluntary and I do not have to take part. My decision whether to be involved will not affect my relationship with the researchers or anyone else at the University of Sydney now or in the future

5.  I understand that my questionnaire responses cannot be withdrawn once they are submitted, as they are anonymous and therefore the researchers will not be able to tell which ones are mine

6.  I understand that personal information about me that is collected over the course of this project will be stored securely and will only be used for purposes that I have agreed to. I understand that information about me will only be told to others with my permission, except as required by law

7.  I understand that the results of the study may be published, and that publications will not contain my name or any identifiable information about me.

Do you consent to the above terms? By answering ‘Yes’, you consent that you have read the Participant Information Sheet and are willing to answer the questions in this survey. If you answer ‘No’, you will not be able to participate in the survey.

- Yes
- No

THE ROLE OF THE GOVERNMENT

Rates of overweight and obesity in Australia have been steadily climbing. In 2014-15, 2 out of 3 adults and 1 in 4 children were classified as overweight or obese. There is growing discussion around the role of the government and the regulatory options available to improve the food environment.

By **government regulation,** we are referring to the introduction and enforcement of laws and standards that aim to protect the health of the population. For example, the Australian Government has introduced laws that prohibit:

- advertising of tobacco products on television and radio
- not wearing seat belts while riding in cars
- alcohol products from being sold to children <18 years of age.

1. How serious do you consider the problem of overweight and obesity in Australia?

- Very serious
- Somewhat serious
- Not too serious
- Not at all serious
- Don’t know

1. How much government regulation do you think there should be around protecting people from overweight and obesity in Australia?

- A great deal
- Some
- A little
- None at all
- Don’t know

REGULATION OF FOOD AND BEVERAGE ADVERTISING

Currently in Australia, the food industry and advertising industry are responsible for regulating what food and beverages can be advertised on television, internet, radio, cinema, print and outdoors. This is referred to as ‘self-regulation’.

Please indicate how strongly you agree or disagree with the following statements:

NOTE: For the purposes of this survey, we are referring to non-alcoholic beverages only.

1. The government should regulate food and beverage advertising in Australia

- Strongly agree
- Somewhat agree
- Neither agree nor disagree
- Somewhat disagree
- Strongly disagree

1. Government regulations should restrict advertising of unhealthy foods and beverages on **television** during times when children (<14 years of age) are watching.

- Strongly agree
- Somewhat agree
- Neither agree nor disagree
- Somewhat disagree
- Strongly disagree

1. Government regulations should restrict advertising of unhealthy foods and beverages to children (<14 years of age) on the **internet**

- Strongly agree
- Somewhat agree
- Neither agree nor disagree
- Somewhat disagree
- Strongly disagree

1. Government regulations should restrict advertising of unhealthy foods and beverages in **public spaces** (e.g. bus stops, train stations, roadside)

- Strongly agree
- Somewhat agree
- Neither agree nor disagree
- Somewhat disagree
- Strongly disagree

1. Government regulations should restrict advertising of unhealthy foods and beverages at **sporting events**

- Strongly agree
- Somewhat agree
- Neither agree nor disagree
- Somewhat disagree
- Strongly disagree

REGULATION OF SPORTS SPONSORSHIPS

Please indicate how strongly you agree or disagree with the following statements:

NOTE: For the purposes of this study, sugar-sweetened beverages refer to soft drinks (not diet or artificially-sweetened), energy drinks, sports drinks, flavoured mineral waters, cordials, sweetened iced tea drinks, and fruit and vegetable drinks. It does not include milk-based products, 100% fruit or vegetable juice, or alcoholic beverages.

1. Government regulations should prohibit **fast food companies** from sponsoring children’s sporting organisations, teams and events

- Strongly agree
- Somewhat agree
- Neither agree nor disagree
- Somewhat disagree
- Strongly disagree

1. Government regulations should prohibit **sugar-sweetened beverage companies** from sponsoring children’s sporting organisations, teams and events

- Strongly agree
- Somewhat agree
- Neither agree nor disagree
- Somewhat disagree
- Strongly disagree

FOOD AND BEVERAGE TAXATION

Please indicate how strongly you agree or disagree with the following statements:

NOTE: For the purposes of this study, unhealthy foods refer to foods high in fat and/or sugar e.g. biscuits, cakes, potato chips, commercial burgers, processed meats, ice cream, confectionary, chocolate.

1. The government should introduce a tax on **unhealthy foods**  to help reduce their consumption

- Strongly agree
- Somewhat agree
- Neither agree nor disagree
- Somewhat disagree
- Strongly disagree

1. The government should introduce a tax on **unhealthy foods**, and use part of the money raised to fund health services and programs to reduce overweight and obesity

- Strongly agree
- Somewhat agree
- Neither agree nor disagree
- Somewhat disagree
- Strongly disagree

1. The government should introduce a tax on **sugar-sweetened beverages** to help reduce their consumption

- Strongly agree
- Somewhat agree
- Neither agree nor disagree
- Somewhat disagree
- Strongly disagree

1. The government should introduce a tax on **sugar-sweetened beverages**, and use part of the money raised to fund health services and programs to reduce overweight and obesity

- Strongly agree
- Somewhat agree
- Neither agree nor disagree
- Somewhat disagree
- Strongly disagree

DEMOGRAPHIC QUESTIONS

1. What is your gender?

- Female
- Male
- Other

1. What is your age?

- 18-24 years
- 25-34 years
- 35-44 years
- 45-54 years
- 55+ years

1. What is your highest level of education?

- Did not complete school
- Year 12 high school
- TAFE/trade/diploma
- University degree or above

1. What is your postcode?

____________

1. What is your weight in kilograms? (estimated if you do not know exactly)

____________

1. What is your height in centimetres? (estimated if you do not know exactly)

_____________

1. Do you have children?

- Yes
- No
- Prefer not to disclose

SURVEY COMPLETED

Thank you for participating in this survey. Once you click ‘Done’ you will be redirected to the Mint Surveys website and will not be able to go back and change your answers.

If you would like to receive feedback about the results of this survey, please contact Emma Sainsbury, Project Officer, on +61 2 8627 2006, or [emma.sainsbury@sydney.edu.au](mailto:emma.sainsbury@sydney.edu.au)
