# Supplementary material for: Public support for government regulatory interventions for overweight and obesity in Australia
Source: BMC Public Health. 2018 Apr 18;18:513. doi: 10.1186/s12889-018-5455-0 (PMC5907362; doi:10.1186/s12889-018-5455-0)
Supplement: Supplementary file 2 — Binomial logistic regression predicting responses to regulatory approaches based on demographic characteristics. (DOCX 61 kb) [file 12889_2018_5455_MOESM2_ESM.docx]

**Additional file 2. Binomial logistic regression predicting responses to regulatory approaches based on demographic characteristics**

|  | **How serious do you consider the problem of overweight and obesity?** | | | **How much government regulation should there be to protect people from overweight and obesity?** | | | **The government should regulate food and beverage advertising** | | |
| --- | --- | --- | --- | --- | --- | --- | --- | --- | --- |
|  | *% very/somewhat* | *OR* | *95% CI* | *% a great deal/some* | *OR* | *95% CI* | *% agree* | *OR* | *95% CI* |
| **Gender** |  |  |  |  |  |  |  |  |  |
| Female | 94.1 | 1.00 (ref) |  | 67.0 | 1.00 (ref) |  | 71.1 | 1.00 (ref) |  |
| Male | 90.9 | 0.46 * | 0.24 - 0.85 | 64.1 | 0.83 | 0.68-1.01 | 67.8 | 0.58 *** | 0.44 - 0.78 |
| **Age** |  |  |  |  |  |  |  |  |  |
| 18-24 years | 90.6 | 1.00 (ref) |  | 73.6 | 1.00 (ref) |  | 72.4 | 1.00 (ref) |  |
| 25-34 years | 92.1 | 2.58 | 0.55 - 12.00 | 68.0 | 0.66 | 0.37 - 1.18 | 71.1 | 1.11 | 0.46 – 2.68 |
| 35-44 years | 91.4 | 1.14 | 0.31 - 4.20 | 66.5 | 0.66 | 0.37 - 1.17 | 69.4 | 0.69 | 0.31 – 1.53 |
| 45-54 years | 91.0 | 0.92 | 0.26 - 3.28 | 65.1 | 0.69 | 0.39 - 1.21 | 65.2 | 0.67 | 0.31 - 1.48 |
| 55+ years | 93.4 | 1.49 | 0.43 - 5.12 | 64.1 | 0.66 | 0.39 - 1.14 | 70.1 | 0.69 | 0.33 – 1.48 |
| **Education level** |  |  |  |  |  |  |  |  |  |
| Did not complete school | 93.0 | 1.00 (ref) |  | 59.6 | 1.00 (ref) |  | 61.2 | 1.00 (ref) |  |
| Year 12 high school | 92.5 | 1.98 | 0.75 – 5.20 | 64.9 | 1.12 | 0.83 - 1.52 | 66.3 | 1.28 | 0.86 – 1.89 |
| TAFE/trade/diploma | 93.5 | 1.49 | 0.70 – 3.17 | 65.5 | 1.19 | 0.92 - 1.55 | 71.5 | 2.25 *** | 1.56 – 3.23 |
| University degree | 90.6 | 1.46 | 0.61 – 3.47 | 72.2 | 1.68 ** | 1.24 - 2.28 | 77.3 | 3.25 *** | 2.08 – 5.07 |
| **Parental status** |  |  |  |  |  |  |  |  |  |
| Children | 94.1 | 1.00 (ref) |  | 65.3 | 1.00 (ref) |  | 70.2 | 1.00 (ref) |  |
| No children | 89.7 | 0.43 * | 0.23 – 0.82 | 67.2 | 1.05 | 0.84 - 1.32 | 68.6 | 0.89 | 0.65 - 1.23 |
| **Socioeconomic status** |  |  |  |  |  |  |  |  |  |
| Tertile 1 | 94.7 | 1.00 (ref) |  | 67.7 | 1.00 (ref) |  | 71.5 | 1.00 (ref) |  |
| Tertile 2 | 91.2 | 0.77 | 0.38 – 1.57 | 62.8 | 0.75 * | 0.60 - 0.95 | 69.1 | 0.65 * | 0.46 - 0.91 |
| Tertile 3 | 91.1 | 0.74 | 0.35 – 1.55 | 66.4 | 0.81 | 0.63 - 1.04 | 67.3 | 0.54 ** | 0.38 - 0.78 |
| **Body Mass Index** |  |  |  |  |  |  |  |  |  |
| Underweight | 95.7 | 1.36 | 0.17 - 10.74 | 60.9 | 0.61 | 0.32 - 1.17 | 85.1 | 2.61 | 0.71 - 9.61 |
| Healthy weight | 91.4 | 1.00 (ref) |  | 68.7 | 1.00 (ref) |  | 69.0 | 1.00 (ref) |  |
| Overweight | 94.2 | 2.03 | 0.87 - 4.74 | 65.8 | 0.87 | 0.68 - 1.12 | 72.1 | 1.51 * | 1.06 - 2.14 |
| Obese | 91.5 | 0.93 | 0.45 - 1.92 | 62.0 | 0.84 | 0.64 - 1.09 | 65.5 | 1.22 | 0.85 - 1.75 |

|  | **Government regulations should restrict advertising of unhealthy foods on television during times when children are watching** | | | **Government regulations should restrict advertising of unhealthy foods to children on the internet** | | | **Government regulations should restrict advertising of unhealthy foods in public spaces** | | |
| --- | --- | --- | --- | --- | --- | --- | --- | --- | --- |
|  | *% agree* | *OR* | *95% CI* | *% agree* | *OR* | *95% CI* | *% agree* | *OR* | *95% CI* |
| **Gender** |  |  |  |  |  |  |  |  |  |
| Female | 82.1 | 1.00 (ref) |  | 78.4 | 1.00 (ref) |  | 72.1 | 1.00 (ref) |  |
| Male | 75.6 | 0.45 *** | 0.32 - 0.63 | 73.5 | 0.52 *** | 0.38 - 0.71 | 68.6 | 0.62 ** | 0.46-0.83 |
| **Age** |  |  |  |  |  |  |  |  |  |
| 18-24 years | 78.3 | 1.00 (ref) |  | 67.6 | 1.00 (ref) |  | 68.9 | 1.00 (ref) |  |
| 25-34 years | 76.4 | 1.08 | 0.43 - 2.70 | 71.5 | 1.81 | 0.84 - 3.88 | 68.0 | 0.99 | 0.45 - 2.16 |
| 35-44 years | 78.5 | 1.33 | 0.55 - 3.22 | 75.1 | 1.94 | 0.93 - 4.04 | 67.7 | 0.87 | 0.41 - 1.83 |
| 45-54 years | 73.2 | 0.84 | 0.36 – 1.95 | 73.0 | 1.61 | 0.80 - 3.24 | 67.2 | 0.90 | 0.436 - 1.89 |
| 55+ years | 81.4 | 1.34 | 0.59 - 3.05 | 78.8 | 2.05 * | 1.05 – 4.00 | 72.8 | 1.10 | 0.54 - 2.21 |
| **Education level** |  |  |  |  |  |  |  |  |  |
| Did not complete school | 70.0 | 1.00 (ref) |  | 70.0 | 1.00 (ref) |  | 63.4 | 1.00 (ref) |  |
| Year 12 high school | 79.8 | 1.44 | 0.92 - 2.26 | 72.7 | 1.31 | 0.84 - 2.04 | 67.6 | 1.10 | 0.72 - 1.67 |
| TAFE/trade/diploma | 81.1 | 2.16 *** | 1.43 – 3.27 | 78.3 | 1.84 ** | 1.24 - 2.73 | 74.0 | 1.64 * | 1.12 - 2.41 |
| University degree | 84.5 | 3.59 *** | 2.14 - 6.03 | 80.9 | 2.76 *** | 1.71 - 4.47 | 74.6 | 2.06 ** | 1.31 - 3.24 |
| **Parental status** |  |  |  |  |  |  |  |  |  |
| Children | 79.7 | 1.00 (ref) |  | 77.7 | 1.00 (ref) |  | 72.4 | 1.00 (ref) |  |
| No children | 77.6 | 1.04 | 0.71 - 1.50 | 73.2 | 0.81 | 0.58 - 1.15 | 66.6 | 0.79 | 0.57 - 1.09 |
| **Socioeconomic status** |  |  |  |  |  |  |  |  |  |
| Tertile 1 | 80.9 | 1.00 (ref) |  | 78.0 | 1.00 (ref) |  | 71.6 | 1.00 (ref) |  |
| Tertile 2 | 77.3 | 0.57 ** | 0.39 - 0.85 | 75.5 | 0.60 ** | 0.41 - 0.87 | 70.8 | 0.69 * | 0.48 – 0.98 |
| Tertile 3 | 77.9 | 0.50** | 0.33 - 0.77 | 73.5 | 0.53 ** | 0.36 - 0.79 | 68.2 | 0.52 ** | 0.36 - 0.76 |
| **Body Mass Index** |  |  |  |  |  |  |  |  |  |
| Underweight | 84.8 | 2.62 | 0.51 - 13.39 | 85.1 | 3.97 | 0.78 - 20.28 | 72.3 | 0.84 | 0.33 - 2.17 |
| Healthy weight | 79.7 | 1.00 (ref) |  | 75.8 | 1.00 (ref) |  | 70.9 | 1.00 (ref) |  |
| Overweight | 79.2 | 1.12 | 0.75 - 1.66 | 78.2 | 1.30 | 0.89 - 1.89 | 70.6 | 0.96 | 0.67 - 1.39 |
| Obese | 76.6 | 1.14 | 0.75 - 1.72 | 72.5 | 1.08 | 0.73 - 1.59 | 68.9 | 0.84 | 0.51 - 1.23 |

|  | **Government regulations should restrict advertising of unhealthy foods at sporting events** | | | **Government regulations should prohibit fast food companies from sponsoring children’s sport** | | | **Government regulations should prohibit SSB companies from sponsoring children’s sport** | | |
| --- | --- | --- | --- | --- | --- | --- | --- | --- | --- |
|  | *% agree* | *OR* | *95% CI* | *% agree* | *OR* | *95% CI* | *% agree* | *OR* | *95% CI* |
| **Gender** |  |  |  |  |  |  |  |  |  |
| Female | 72.4 | 1.00 (ref) |  | 62.3 | 1.00 (ref) |  | 66.9 | 1.00 (ref) |  |
| Male | 70.4 | 0.69 * | 0.52 - 0.93 | 55.2 | 0.65 *** | 0.51 - 0.83 | 59.9 | 0.68 ** | 0.53 - 0.87 |
| **Age** |  |  |  |  |  |  |  |  |  |
| 18-24 years | 69.5 | 1.00 (ref) |  | 52.8 | 1.00 (ref) |  | 57.5 | 1.00 (ref) |  |
| 25-34 years | 68.9 | 1.36 | 0.63 - 2.91 | 62.4 | 1.96 * | 1.05 - 3.65 | 64.5 | 1.54 | 0.80 - 2.96 |
| 35-44 years | 67.0 | 1.13 | 0.56 - 2.31 | 58.0 | 2.17 * | 1.18 - 3.98 | 63.3 | 2.02 * | 1.06 - 3.84 |
| 45-54 years | 67.9 | 1.20 | 0.59 - 2.43 | 58.6 | 1.84 * | 1.02 - 3.33 | 63.0 | 1.58 | 0.85 - 2.94 |
| 55+ years | 74.6 | 1.42 | 0.73 - 2.77 | 58.7 | 2.01 * | 1.14 – 3.54 | 64.0 | 1.77 | 0.97 - 3.22 |
| **Education level** |  |  |  |  |  |  |  |  |  |
| Did not complete school | 65.0 | 1.00 (ref) |  | 49.1 | 1.00 (ref) |  | 53.6 | 1.00 (ref) |  |
| Year 12 high school | 70.7 | 1.07 | 0.70 - 1.63 | 56.6 | 1.37 | 1.00 - 2.06 | 61.9 | 1.32 | 0.92 - 1.86 |
| TAFE/trade/diploma | 73.4 | 1.52 * | 1.04 - 2.22 | 61.1 | 1.78 *** | 1.35 - 2.54 | 66.1 | 1.73 ** | 1.26 - 2.36 |
| University degree | 75.5 | 1.97 ** | 1.26 - 3.26 | 66.8 | 2.26 *** | 1.58 - 3.28 | 70.8 | 2.48 *** | 1.69 - 3.63 |
| **Parental status** |  |  |  |  |  |  |  |  |  |
| Children | 73.4 | 1.00 (ref) |  | 60.0 | 1.00 (ref) |  | 64.8 | 1.00 (ref) |  |
| No children | 67.7 | 0.88 | 0.63 - 1.22 | 57.3 | 1.09 | 0.83 - 1.46 | 61.4 | 1.04 | 0.78 - 1.38 |
| **Socioeconomic status** |  |  |  |  |  |  |  |  |  |
| Tertile 1 | 74.0 | 1.00 (ref) |  | 58.1 | 1.00 (ref) |  | 62.9 | 1.00 (ref) |  |
| Tertile 2 | 70.3 | 0.66 * | 0.47 - 0.94 | 58.9 | 1.02 | 0.77 - 1.36 | 63.8 | 0.99 | 0.75 - 1.32 |
| Tertile 3 | 69.5 | 0.50 *** | 0.34 - 0.71 | 59.3 | 0.80 | 0.60 - 1.09 | 63.6 | 0.82 | 0.61 - 1.12 |
| **Body Mass Index** |  |  |  |  |  |  |  |  |  |
| Underweight | 82.6 | 1.06 | 0.40 - 2.82 | 47.8 | 0.61 | 0.27 - 1.38 | 59.6 | 0.89 | 0.36 - 2.19 |
| Healthy weight | 72.0 | 1.00 (ref) |  | 62.2 | 1.00 (ref) |  | 66.7 | 1.00 (ref) |  |
| Overweight | 73.7 | 0.88 | 0.62 - 1.26 | 61.0 | 0.88 | 0.66 - 1.19 | 64.9 | 0.83 | 0.61 - 1.12 |
| Obese | 67.6 | 0.78 | 0.53 - 1.13 | 53.8 | 0.78 | 0.58 - 1.08 | 58.2 | 0.72 * | 0.52 - 0.99 |

|  | **The government should introduce a tax on unhealthy foods** | | | **The government should introduce a tax on unhealthy foods, and use the money for health purposes** | | | **The government should introduce a tax on SSBs** | | |
| --- | --- | --- | --- | --- | --- | --- | --- | --- | --- |
|  | *% agree* | *OR* | *95% CI* | *% agree* | *OR* | *95% CI* | *% agree* | *OR* | *95% CI* |
| **Gender** |  |  |  |  |  |  |  |  |  |
| Female | 50.6 | 1.00 (ref) |  | 55.3 | 1.00 (ref) |  | 56.0 | 1.00 (ref) |  |
| Male | 47.3 | 0.82 | 0.67 - 1.02 | 53.8 | 0.86 | 0.70 - 1.07 | 53.2 | 0.73 ** | 0.59 - 0.91 |
| **Age** |  |  |  |  |  |  |  |  |  |
| 18-24 years | 53.3 | 1.00 (ref) |  | 67.0 | 1.00 (ref) |  | 59.0 | 1.00 (ref) |  |
| 25-34 years | 57.9 | 0.92 | 0.51 - 1.65 | 57.0 | 0.42 * | 0.22 - 0.83 | 57.5 | 0.62 | 0.33 - 1.18 |
| 35-44 years | 49.5 | 0.71 | 0.40 - 1.26 | 52.9 | 0.39 ** | 0.20 - 0.75 | 55.2 | 0.59 | 0.32 - 1.11 |
| 45-54 years | 39.3 | 0.46 ** | 0.26 - 0.81 | 46.8 | 0.27 *** | 0.14 - 0.52 | 440 | 0.37 ** | 0.20 - 0.69 |
| 55+ years | 49.4 | 0.79 | 0.46 - 1.36 | 55.7 | 0.43 ** | 0.23 - 0.82 | 56.7 | 0.71 | 0.39 - 1.30 |
| **Education level** |  |  |  |  |  |  |  |  |  |
| Did not complete school | 38.8 | 1.00 (ref) |  | 44.7 | 1.00 (ref) |  | 45.9 | 1.00 (ref) |  |
| Year 12 high school | 45.9 | 1.34 | 0.98 - 1.85 | 53.5 | 1.36 | 0.98 - 1.88 | 51.8 | 1.27 | 0.93 - 1.75 |
| TAFE/trade/diploma | 50.8 | 1.61 ** | 1.22 - 2.11 | 56.5 | 1.60 ** | 1.21 - 2.11 | 55.4 | 1.53 ** | 1.17 - 2.01 |
| University degree | 59.0 | 2.64 *** | 1.91 - 3.65 | 62.6 | 2.27 *** | 1.63 - 3.15 | 63.9 | 2.53 *** | 1.83 - 3.51 |
| **Parental status** |  |  |  |  |  |  |  |  |  |
| Children | 50.1 | 1.00 (ref) |  | 56.5 | 1.00 (ref) |  | 55.8 | 1.00 (ref) |  |
| No children | 47.0 | 0.78 * | 0.62 - 0.99 | 51.3 | 0.77 * | 0.60 - 0.98 | 52.1 | 0.87 | 0.69 - 1.11 |
| **Socioeconomic status** |  |  |  |  |  |  |  |  |  |
| Tertile 1 | 47.6 | 1.00 (ref) |  | 52.3 | 1.00 (ref) |  | 53.2 | 1.00 (ref) |  |
| Tertile 2 | 47.8 | 1.09 | 0.85 - 1.39 | 53.7 | 1.11 | 0.86 - 1.43 | 52.7 | 1.06 | 0.83 - 1.36 |
| Tertile 3 | 52.0 | 1.04 | 0.80 - 1.35 | 58.5 | 1.15 | 0.88 - 1.50 | 58.5 | 1.11 | 0.85 - 1.45 |
| **Body Mass Index** |  |  |  |  |  |  |  |  |  |
| Underweight | 44.7 | 0.64 | 0.32 - 1.30 | 55.6 | 0.69 | 0.34 - 1.41 | 47.8 | 0.51 | 0.25-1.02 |
| Healthy weight | 53.1 | 1.00 (ref) |  | 56.8 | 1.00 (ref) |  | 58.0 | 1.00 (ref) |  |
| Overweight | 48.5 | 0.89 | 0.69 - 1.15 | 53.7 | 0.85 | 0.66 - 1.12 | 55.4 | 0.86 | 0.66 - 1.12 |
| Obese | 43.1 | 0.85 | 0.65 - 1.13 | 51.2 | 0.96 | 0.72 - 1.28 | 49.5 | 0.89 | 0.67 - 1.18 |

|  | **The government should introduce a tax on SSBs, and use the money for health purposes** | | |
| --- | --- | --- | --- |
|  | *% agree* | *OR* | *95% CI* |
| **Gender** |  |  |  |
| Female | 58.3 | 1.00 (ref) |  |
| Male | 56.0 | 0.81 | 0.65 – 1.01 |
| **Age** |  |  |  |
| 18-24 years | 72.6 | 1.00 (ref) |  |
| 25-34 years | 57.6 | 0.35 ** | 0.17 - 0.71 |
| 35-44 years | 54.8 | 0.33 ** | 0.16 - 0.66 |
| 45-54 years | 48.2 | 0.24 *** | 0.12 - 0.47 |
| 55+ years | 59.0 | 0.39 ** | 0.20 - 0.77 |
| **Education level** |  |  |  |
| Did not complete school | 45.9 | 1.00 (ref) |  |
| Year 12 high school | 57.7 | 1.56 ** | 1.12 - 2.17 |
| TAFE/trade/diploma | 59.2 | 1.67 *** | 1.27 - 2.21 |
| University degree | 65.3 | 2.74 *** | 1.95 - 3.84 |
| **Parental status** |  |  |  |
| Children | 58.6 | 1.00 (ref) |  |
| No children | 55.0 | 0.79 | 0.62 - 1.01 |
| **Socioeconomic status** |  |  |  |
| Tertile 1 | 54.4 | 1.00 (ref) |  |
| Tertile 2 | 56.2 | 1.10 | 0.86 - 1.42 |
| Tertile 3 | 61.6 | 1.18 | 0.90 - 1.55 |
| **Body Mass Index** |  |  |  |
| Underweight | 55.3 | 0.72 | 0.34 - 1.50 |
| Healthy weight | 58.9 | 1.00 (ref) |  |
| Overweight | 58.5 | 0.96 | 0.73 - 1.25 |
| Obese | 53.8 | 1.07 | 0.80 - 1.43 |

OR = odds ratio; CI = confidence interval; (ref) = reference category for odds ratio analysis
Results from binomial logistic regression with all variables included in the model. Models are based on data weighted for education level.
The “% very/somewhat” category represents respondents who indicated they consider the problem of overweight or obesity to be “very serious” or “somewhat serious”
The “% a great deal/some” category represents respondents who indicated there should be “a great deal” or “some” government regulation to protect people from overweight and obesity
The “% agree” category represents respondents who “somewhat agree” or “strongly agree” with the proposed regulatory measures.
The p-values (* p<0.05, ** p<0.01, *** p<0.001) shown on the table reflect statistically significant differences between the reference category and the other categories in each variable.
